# THE EFFECTS OF VISUAL SEARCH ON POSTURAL SWAY COMPLEXITY IN OLDER ADULTS WITH AND WITHOUT TYPE 2 DIABETES

**DOI:** 10.1093/geroni/igac059.2910

**Published:** 2022-12-20

**Authors:** Kellyn Jason, Junhong Zhou, Wanting Yu, Brad Manor, Azizah Jor’dan

**Affiliations:** University of Massachusetts Boston, Boston, Massachusetts, United States; Harvard Medical School/Hebrew SeniorLife, Roslindale, Massachusetts, United States; Hebrew SeniorLife, Roslindale, Massachusetts, United States; Hebrew SeniorLife/Harvard Medical School, Roslindale, Massachusetts, United States; University of Massachusetts, Boston, Massachusetts, United States

## Abstract

Type II diabetes mellitus (T2DM) is linked to impairments in the interconnecting and interacting components (e.g., cognitive-motor demand) essential for standing postural control. The temporal dynamics of postural sway are complex, thus higher complexity reflects the system’s capacity to adapt to cognitive-motor demands. We aimed to characterize the effects of T2DM on postural sway complexity during visual search tasks (VST). Twenty-four older adults without T2DM (OA) (age=67–93 years) and 20 older adults with T2DM (age=70–90 years) performed multiple trials of quiet standing with and without a VST. The VST consisted of low difficulty (LD) (i.e., counting the frequency of one designated letter in a random grid of letters) and high difficulty (HD) (i.e., counting two different letters). The complexity of the postural sway acceleration signals in the anterior-posterior (AP) and medial-lateral (ML) directions were quantified using multiscale entropy. Across participants, matched-pairs analyses revealed that both AP and ML sway complexity increased during the LD condition, compared to control (p < 0.007). During HD, only the AP sway complexity increased, compared to control (p=0.0001). Within the OA, both AP and ML sway complexity increased during the LD condition (p < 0.009), while performance of the HD task showed an increase in AP sway complexity compared to control (p=0.0002). Within the T2DM, AP sway complexity increased only during LD, compared to control (p=0.03). The multi-scale dynamics of postural control when standing while performing VSTs differ among older adults with and without T2DM, reflected by diminished capacity to increase complexity during the visual search conditions.

